# Elevated Plasma Levels of Interleukin-12p40 and Interleukin-16 in Overweight Adolescents

**DOI:** 10.1155/2015/940910

**Published:** 2015-02-02

**Authors:** Michael Lichtenauer, Marcus Franz, Michael Fritzenwanger, Hans-Reiner Figulla, Norbert Gerdes, Christian Jung

**Affiliations:** ^1^Department of Cardiology, Clinic of Internal Medicine II, Paracelsus Medical University of Salzburg, Müllner Hauptstraße 48, 5020 Salzburg, Austria; ^2^Department of Cardiology, Clinic of Internal Medicine I, Friedrich Schiller University Jena, Erlanger Allee 101, 07747 Jena, Germany; ^3^Ludwig Maximilian University of Munich, Pettenkoferstrasse 8a, 80336 Munich, Germany

## Abstract

*Introduction*. Obesity during adolescence is an increasing problem for both the individual and health care systems alike. In Western world countries, childhood adiposity has reached epidemic proportions. It is known that elevated levels of proinflammatory cytokines can be found in the plasma of obese patients. In this study, we sought to determine the relation between IL-12p40, IL-12p70, and Interleukin-16 (IL-16) in overweight adolescents. *Materials and Methods*. Seventy-nine male Caucasian adolescents aged 13–17 years were included in this study. Thirty-seven of them had a body mass index (BMI) above the 90th age-specific percentile. Il-12p40, IL-12p70, and IL-16 were measured from plasma using Luminex multiplex technology. *Results*. Both IL-12p40 and IL-16 concentrations were significantly increased in overweight subjects compared to normal weight controls (IL-12p40: 1086.6 pg/mL ± 31.7 pg/mL SEM versus 1228.6 pg/mL ± 43.5 pg/mL SEM; IL-16 494.0 pg/mL ± 29.4 pg/mL SEM versus 686.6 pg/mL ± 52.5 pg/mL SEM, *P* < 0.05 and *P* < 0.01, resp.). No differences were found for IL-12p70. *Conclusions*. Based on these results, we believe that the increased levels of IL-12p40 and IL-16 are associated with an ongoing inflammatory response in obese individuals and could lead to the development of disease conditions related to obesity.

## 1. Introduction

The development of obesity in children and adolescents has reached almost epidemic proportions in industrialized countries of the Western world since 1980. More than a fifth of all 12–19 year olds in the United States in 2012 were considered obese [1]. Adolescent adiposity is also closely related to persistent obesity in adult patients [2]. A plethora of studies has shown that higher body mass index (BMI) or other measures of obesity in children and adolescents are associated with adverse alterations of lipoprotein ratio, hypertension, insulin resistance, and diabetes [3, 4]. Moreover, abdominal obesity is also the hallmark for the metabolic syndrome (MS) as excess body weight is accompanied with hypertension, elevated blood glucose levels, and hyperlipidemia. Obesity also impairs the outcome in many chronic autoimmune diseases such as asthma.

The high prevalence of obesity in most Western countries also represents immense costs for health care systems. US studies showed that obesity in children is accompanied with higher hospitalization rates for comorbidities of obesity and drug prescription costs. Furthermore, it was shown that an elevated BMI was associated with 14.1 billion US dollars only for nonhospitalization expenditures [5].

Research of recent years showed that smoldering inflammatory processes in adipose tissue are present in overweight patients. Also in children, obesity was accompanied with higher concentrations of C-reactive protein indicating a low grade inflammatory state in these subjects [6].

This ongoing inflammation even at a low level could serve as an elicitor for endothelial stress reactions during the development of atherosclerosis over the years. This collectivization of permanent inflammation and endothelial dysfunction is causative for several cardiovascular pathologies such as coronary heart disease or hypertension which are more likely to occur in obese patients. A vast number of studies have investigated proinflammatory mediators like tumor-necrosis factor (TNF) or Interleukin-6 in adipose patients. However, the signaling axis via Interleukin-12 (IL-12) and Interleukin-16 (IL-16) has not yet received much attention in this context.

Here, we tried to examine the association of IL-12p40, IL-12p70, and IL-16 with obesity in a study cohort of overweight adolescents.

IL-12 is a major factor in early inflammatory responses and in the generation of T-helper type 1 (Th1) cells [7]. Elevated levels of IL-12 were thought to be related to the pathogenesis of autoimmune diseases associated with inflammatory reactions such as multiple sclerosis, arthritis, and insulin dependent diabetes [8–12]. Il-12 consists of a heavy chain (p40 subunit) and a light chain (p35 subunit) which are linked covalently by disulfide bonds to form the p70 molecule [13, 14]. Moreover, dendritic cells and macrophages were also shown to secrete the p40 subunit. The p40 subunit can also serve as a chemoattractant for macrophages [15] and dendritic cells [16] and induces the secretion of TNF-alpha and IL-16 in microglia cells and macrophages [17].

IL-16, also known as a leukocyte chemoattractant factor (LCF), is considered a proinflammatory cytokine and is produced by T-cells but also neutrophils and monocytes. It could promote migration of lymphocytes, induce the expression of proinflammatory factors, and modulate apoptosis. Moreover, peripheral blood mononuclear cells (PBMC), monocytes, and macrophages, but not CD4+ T-cells, were shown to secrete Interleukin-1beta, IL-6, and TNF-alpha upon stimulation with IL-16 [18].

The aim of this study was to investigate IL-12p40, IL-12p70, and IL-16 in overweight adolescents and their association with anthropometrical measurements of obesity.

## 2. Methods

### 2.1. Study Subjects

The experimental protocol for this study was approved by the local ethics committee (Friedrich-Schiller-University, Jena, Germany). All study subjects and their parents were provided with information on the course of the study and written informed consent was obtained. All experiments were performed in accordance with the principles of the Declaration of Helsinki and Good Clinical Practice. In total, 79 voluntary male individuals aged 13–17 years going to schools in the area of greater Jena were included in this study. Of these, 37 (46%) had a body mass index (BMI) above the 90th percentile according to German charts [19]. After receipt of informed consent, the following parameters were recorded in one consultation: age, body height, body weight, heart rate, systolic and diastolic blood pressure, BMI, waist circumference, exercise rate (more or less than 3 hours per week), and family history of diabetes or cardiovascular disease. Waist circumference was measured as described previously [20]. In brief, waist circumference was measured during minimal respiration to the nearest 0.1 cm at the level of the iliac crest.

### 2.2. Blood Samples

All blood samples were collected by venipuncture from an antecubital vein using heparin and EDTA anticoagulated tubes in the morning after an overnight fast. The tubes were immediately processed and centrifuged within 60 minutes after blood withdrawal. Standard plasma parameters were obtained from the Department of Clinical Chemistry at the University Hospital Jena: high-density lipoprotein (HDL; mmol/L), low-density lipoprotein (LDL; mmol/L), triglycerides (mmol/L), C-reactive protein (CRP, mg/L; high sensitivity assay), and hematological parameters (e.g., counts for leukocytes and erythrocytes). Furthermore, aliquots of obtained plasma samples were stored at −80° until cytokine measurements were conducted.

### 2.3. Cytokine Measurements

The quantitative determination of human TNF-alpha, IL-6, IL-10, IL-12p40, IL-12p70, and IL-16 was performed from immediately frozen heparin plasma (−80° Celsius). Cytokine concentrations were determined using Bio-Plex technology according to the manufacturer's instructions (Bio-Rad, Hercules, USA). Summarily, fluorescently dyed microspheres coated with capture antibodies bind to relevant cytokines in a sandwich immunoassay format. Unbound sample is removed in a washing step and a specific protein can be detected with a fluorescently labeled antibody. Quantitative measurements of cytokine levels were performed in a Luminex analyzer as previously described [21].

### 2.4. Statistical Analysis

Statistical analysis was performed using SPPS (21.0, SPSS Inc., USA) and GraphPad Prism software (GraphPad Software, USA). All data are given as mean ± standard error of the mean (SEM). IL-12p40, IL-12p70, and IL-16 concentrations between groups were compared using the Mann-Whitney *U* test. Baseline characteristics were compared using the two-sided Student's *t*-test. In order to further explore the association between cytokines and patient characteristics, correlation analysis was performed (using Spearman correlation coefficient). The Bonferroni-Holm method was used to correct for multiple testing. *P* values < 0.05 were considered statistically significant. A *P* value of <0.05 was indicated as ∗ and a *P* of < 0.01 as ∗∗.

## 3. Results

Baseline characteristics for all 79 participants in this study are presented in Table 1 (see also [22–25]). Significant differences were found for weight, BMI, waist circumference, systolic blood pressure, erythrocytes, MCV, MCH, thrombocytes, monocytes, high sensitive C-reactive protein, HbA1c, creatinine, and high-density lipoprotein. As expected, overweight adolescents evidenced higher body weight, BMI, waist circumference, and lower high-density lipoprotein. Moreover, overweight subjects also showed higher levels of C-reactive protein indicating low grade inflammation.

**Figure 1:**
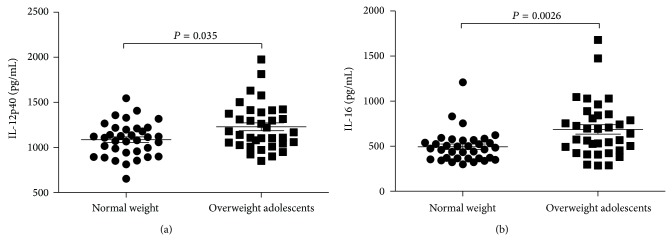
Plasma levels of IL-12p40 (a) and IL-16 (b) in overweight and normal weight adolescents. Data is shown as mean ± standard error of the mean, *n* = 79.

Plasma concentration for both IL-12p40 and IL-16 was also significantly increased in the overweight group (Figure 1). In the control group, IL-12p40 was found at a concentration of 1086.6 pg/mL (±31.7 pg/mL SEM). IL-16 levels were 494.0 pg/mL (±29.4 pg/mL SEM). In overweight adolescents, IL-12p40 was increased to 1228.6 pg/mL (±43.5 pg/mL SEM, *P* = 0.035) and IL-16 to 686.6 pg/mL (±52.5 pg/mL SEM, *P* = 0.0026). No differences were found for IL-12p70 (7.48 pg/mL ± 0.72 pg/mL SEM versus 7.20 ± 1.33 pg/mL SEM, *P* = 0.86). Also no significant differences were found for TNF-alpha, IL-6, and IL-10 between the groups.

**Figure 2:**
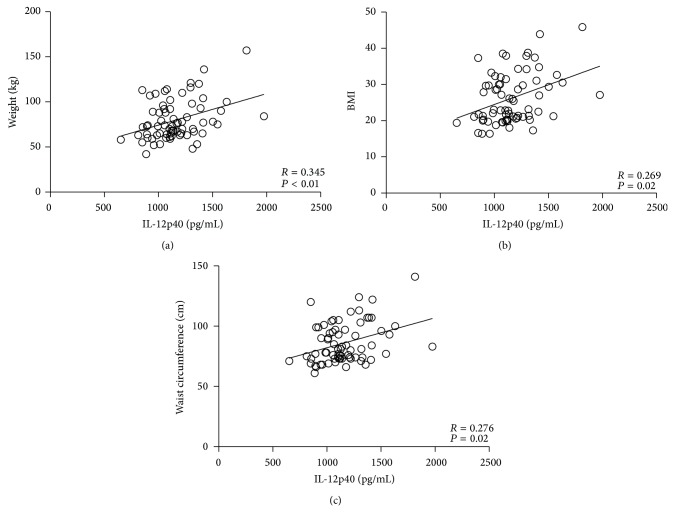
Association (correlation analysis) for IL-12p40 with weight (a), BMI (b), and waist circumference (c).

**Figure 3:**
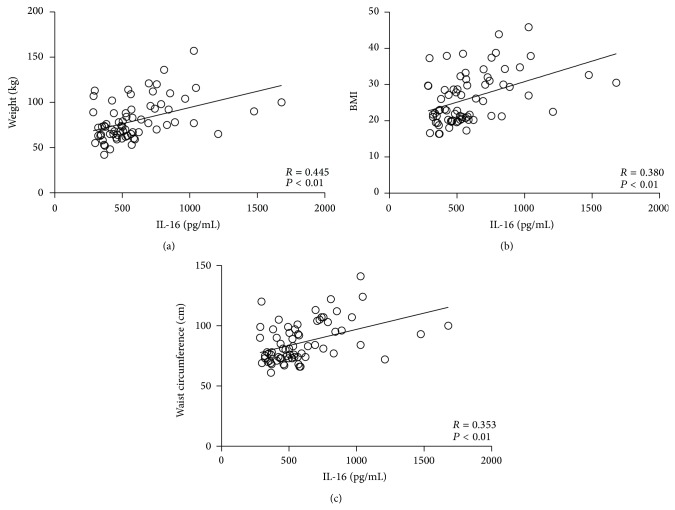
Correlation analysis for the association between IL-16 and weight (a), BMI (b), and waist circumference (c).

In order to further explore the association of IL-12p40 and IL-16 with subject parameters, we performed a correlation analysis. Correlations between clinical characteristics and IL-12p40 and IL-16 are depicted in Figures 2 and 3. IL-12p40 and IL-16 correlated significantly with anthropometrical parameters of obesity (body weight, BMI, and waist circumference). Of interest was that also a strong correlation to inflammatory and hematological parameters was found (Table 2). IL-12p70 did not correlate with baseline parameters. Moreover, the exercise rate of participating subjects did not correlate with IL-12p40 or IL-16 levels.

## 4. Discussion

Obesity strongly contributes to the development of cardiovascular pathologies and other morbidities such as diabetes. Primarily in Western world countries, the incidence of obesity is increasing over the last decades leading to many socioeconomic problems.

In obese patients, low grade inflammation is present as higher levels of proinflammatory cytokines were found in these individuals. With this current study, we aimed to expand the knowledge on cytokine regulation in obese adolescents with the main focus on the IL-12/IL-16 axis. As previous studies showed that IL-12p40 leads to increased secretion of IL-16 [17] which in turn further induced the release of many proinflammatory cytokines such as TNF-alpha and IL-6 from mononuclear cells, we believed that the regulatory mechanism between IL-12 and IL-16 signaling could be of greater importance in adipose children and adults.

We found that significantly higher concentrations of IL-12p40 and IL-16 but not IL-12p70 are present in the plasma of overweight adolescents. Levels of IL-12p40 and IL-16 correlated also significantly with anthropometrical measurements of obesity such as weight, BMI, and waist circumference. In this current study, we also analyzed serum concentrations of other cytokines, such as TNF-alpha, IL-6, and IL-10. However, levels of these cytokines did not differ significantly between groups.

Interestingly, relevant correlations were found when comparing levels of IL-12p40 and IL-16 with hematological parameters (leukocytes and neutrophils but not monocytes and lymphocytes). This could relate to the fact that slightly higher white blood cell counts were found in obese subjects. We hypothesized that the state of permanent low grade inflammation could be causative for these elevated levels of immune cells in the peripheral circulation as also higher levels of C-reactive protein were found. As IL-16 is mainly secreted by neutrophils, monocytes, dendritic cells [26], and CD8+ T-cells [27, 28], the higher number of these cell types could be also associated with the higher concentrations of IL-16 in the plasma of overweight probands. In one of our previous studies, we have shown that IL-16 is secreted in upon activation induced cell death by PBMC after coincubation with antithymocyte globulin [29]. IL-16 can cause the release of other proinflammatory cytokines and the process of ongoing inflammation is further substantiated in overweight patients subsequently leading to the progression of pathologies associated with adiposity.

Moreover, IL-16 is also involved in many autoimmune disease conditions [30] such as inflammatory bowel disease [31], rheumatoid arthritis [32], and airway hyperresponsiveness [33]. Many previous studies have determined a connection between childhood obesity and asthma [34, 35]. Obesity also increases the risk to develop rheumatoid arthritis [36]. In a mouse model of high-fat diet-induced obesity, it was shown that adiposity could lead to exacerbation of inflammatory bowel disease [37].

Interestingly, other prominent inflammatory mediators such as TNF-alpha and IL-6 showed no significant differences between the two study groups. One could speculate that the IL-12p40/IL-16 axis might be a more forefront proinflammatory signaling pathway in the earlier stages of obesity related disease conditions as they were likely present in our cohort of adolescents compared to the inflammatory milieu found in adult patients.

## 5. Limitations

Previous studies showed that IL-16 is upregulated within the adipose tissue of obese mice [38]. Based on our results, we cannot definitively distinguish the main origin of IL-16 as we only analyzed serum samples and not tissue samples of study subjects. Further studies are warranted in order to decode mechanisms leading to the expression of IL-12p40, IL-16, and other inflammatory factors in the context of obesity. It would be of great interest to identify cell types being the main source for the proinflammatory response in adipose patients. This knowledge would be of considerable importance to provide a better understanding of pathophysiological mechanisms induced by low grade inflammation and might help to develop new treatment strategies to counteract disease progression.

## 6. Conclusion

In summary, we conclude that the heightened levels of cytokines of the IL-12/IL-16 axis are involved in pathophysiological conditions that are found in overweight adolescents. Notably, IL-12p40 and IL-16 but not IL-12p70 are elevated in overweight adolescents, correlating with different anthropometrical parameters of obesity. Increased levels of these mediators could lead to the progression of diseases associated with obesity and the metabolic syndrome.

## Figures and Tables

**Table 1:** Demographic characteristics.

	Normal weight	Overweight adolescents	Significance
*n*	42	37	
Age	15.4 ± 0.9	15.0 ± 1.4	ns
Height (cm)	176 ± 8	174 ± 9	ns
Weight (kg)	63 ± 7.6	98 ± 19.1	<0.001
BMI	20.48 ± 1.80	32.43 ± 5.20	<0.001
Waist circumference (cm)	73 ± 5	101 ± 13	<0.001
Heart rate (per minute)	78 ± 17	78 ± 11	ns
Systolic blood pressure (mmHg)	120 ± 11	134 ± 17	0.008
Diastolic blood pressure (mmHg)	72 ± 9	72 ± 9	ns
Leukocytes (×10^9^/L)	6.4 ± 1.6	6.6 ± 1.8	ns
Erythrocytes (×10^12^/L)	5.2 ± 0.3	5.3 ± 0.4	0.042
Hemoglobin (mmol/L)	9.4 ± 0.5	9.2 ± 0.7	ns
Hematocrit	0.44 ± 0.02	0.43 ± 0.03	ns
MCV (fL)	85 ± 3	81 ± 4	<0.001
MCH (fmol)	1.82 ± 0.06	1.74 ± 0.10	<0.001
MCHC (mmol/L)	21.50 ± 0.36	21.57 ± 0.58	ns
Thrombocytes (×10^9^/L)	244 ± 54	269 ± 38	0.02
Neutrophils (×10^9^/L)	3.34 ± 1.46	3.46 ± 1.40	ns
Monocytes (×10^9^/L)	0.48 ± 0.14	0.58 ± 0.14	0.003
Lymphocytes (×10^9^/L)	2.35 ± 0.55	2.34 ± 0.65	ns
HbA1c (%)	5.04 ± 0.28	5.27 ± 0.25	<0.001
Creatinine (*μ*mol/L)	84.71 ± 8.46	73.97 ± 12.13	<0.001
Cholesterol (mmol/L)	4.01 ± 0.80	4.08 ± 0.75	ns
High-density lipoprotein (mmol/L)	1.29 ± 0.22	1.09 ± 0.22	<0.001
Low-density lipoprotein (mmol/L)	2.44 ± 0.83	2.55 ± 0.70	ns
Triglycerides (mmol/L)	0.98 ± 0.56	1.12 ± 0.76	ns
High sensitive C-reactive protein (mg/L)	0.52 ± 1.37	2.95 ± 3.51	<0.001
TNF-alpha (pg/mL)	69.65 ± 5.21	62.32 ± 4.73	ns
IL-6 (pg/mL)	7.01 ± 0.82	7.48 ± 0.96	ns
IL-10 (pg/mL)	13.21 ± 1.86	18.62 ± 4.47	ns

**Table 2:** Correlation of IL-12p40 and IL-16 with lab parameters, indicated as Spearman correlation coefficient (*R*).

	IL-12p40	IL-16
Leukocytes (×10^9^/L)	0.305^**^	0.286^*^
Neutrophils (×10^9^/L)	0.210ns	0.285^*^
Monocytes (×10^9^/L)	−0.121ns	−0.092ns
Lymphocytes (×10^9^/L)	−0.021ns	−0.137ns
High sensitive C-reactive protein (mg/L)	0.413^**^	0.480^**^

ns: not significant.

^*^ *P* < 0.05.

^**^ *P* < 0.01.
